# A prospective clinical investigation of the effects of anti-TNF alpha therapy on exercise capacity in patients with ankylosing spondylitis

**DOI:** 10.3906/sag-1805-291

**Published:** 2019-02-11

**Authors:** Erhan ÇAPKIN, Seyhan Bilge KESKİN, Murat KARKUCAK, Ahmet AYAR

**Affiliations:** 1 Division of Rheumatology, Department of Physical Medicine and Rehabilitation, School of Medicine, Farabi Hospital,Karadeniz Technical University, Trabzon Turkey; 2 Department of Physical Medicine and Rehabilitation, School of Medicine, Farabi Hospital,Karadeniz Technical University, Trabzon Turkey; 3 Department of Physiology, School of Medicine, Karadeniz Technical University, Trabzon Turkey

**Keywords:** Ankylosing spondylitis, anti-TNF alpha therapy, aerobic capacity, physical functional capacity

## Abstract

**Background/aim:**

The purpose of this study was to investigate possible effects of anti-TNF alpha therapy on cardiorespiratory fitness and physical functional capacity of patients with ankylosing spondylitis (AS).

**Materials and methods:**

Twenty-eight AS patients meeting the modified New York criteria with active disease state and an equivalent number of healthy individuals as the control were prospectively enrolled. Physical working capacity and aerobic exercise capacity of the participants were determined by using cardiopulmonary exercise tests, performed before and 4 months after initiation of anti-TNF alpha therapy.

**Results:**

The mean age of the patients was 37 ± 9.1 years, and mean duration of disease was 8.9 ± 7.6 years. Patients with AS exhibited significantly lower aerobic exercise capacity (VO2peak: 21.2 ± 5.5 vs. 27.2 ± 6.6 ml/kg/min, P = 0.001), maximum power output (110.4 ± 34.8 vs. 153 ± 39.8 W, P = 0.0001), and exercise duration (16.3 ± 2.6 vs. 19.6 ± 2.9 min, P = 0.0001) than the healthy controls. When patients were reevaluated after 4 months of anti-TNF alpha therapy, significant improvement was obtained in patients’ aerobic capacity, maximum power output, and exercise duration.

**Conclusion:**

Results from this study indicate that in addition to inflammatory parameters and quality of life index, even short-term anti-TNF alpha therapy results in significant improvement in cardiopulmonary health status as objectively reflected by peak VO2, maximum work rate, and exercise duration.

## 1. Introduction

Ankylosing spondylitis (AS) is a chronic inflammatory disease involving mainly the sacroiliac joints and the spine (1). AS may show extraarticular manifestations, including cardiovascular and pulmonary involvements (2). In conjunction with systemic inflammation, extraarticular involvement can seriously impair exercise capacity and overall quality of life of patients. Physical functioning in AS is independently determined by both disease activity and radiographic damage of the spine. AS can also lead to significant exercise intolerance (3). AS-associated comorbidities show a significant correlation with the disease activity index (4,5). By investigating the levels of fatigue in patients with AS, Dagfinrud et al. determined significantly more fatigue, with about one out of three reporting serious levels of fatigue, compared with the general population (6). Pain, mobility restrictions, sleep disturbance, and fatigue are among the common problems causing poor quality of life in patients with AS. Thus, given the early age of onset, the chronic and progressive nature of pain, severe deformity, and impairment of life quality and work capacity are serious issues in AS with profound effects on individuals and society on a large scale (7–10). The primary aim of current AS therapy is to relieve symptoms as well as maintain functionality, improve quality of life, and prevent structural damage(s). To achieve these goals, pharmacological and nonpharmacological interventions are utilized in combination. Antitumor necrosis factor (TNF) alpha agents are currently the best medical therapy choice for AS. These agents not only provide improvement in clinical signs and symptoms but also improve physical functioning capacity and overall quality of life of patients. Therapy with anti-TNF alpha agents restores acute phase response and decreases inflammation scores on magnetic resonance imaging (MRI) evaluation. Improved self-reported physical functioning and quality of life, although subjective, has been reported by AS patients after treatment with anti-TNF alpha agents (5,11). 

Cardiopulmonary exercise testing (CPET) is an objective, reliable noninvasive laboratory test being increasingly used in a wide spectrum of research and clinical applications. It allows comprehensive evaluation of individuals’ functional capacity by linking the pulmonary, cardiovascular, and metabolic responses to exercise. With performance of the symptom-limited maximum level of physical exercise, CPET reveals individuals’ functional capacity, exercise capacity, and exercise limits that cannot be detected clinically and yet are less pronounced at rest. CPET entails measurements of a broad range of variables related to cardiorespiratory function, including oxygen uptake (VO2), carbon dioxide output (VCO2), and minute ventilation (VE) along with electrocardiography (ECG), blood pressure, and pulse oximetry. By gathering these data during maximal symptom-limited incremental exercise testing, CPET allows objective evaluation of exercise performance and functional capacity, and thereby the overall health of individuals at baseline and in response to medical or surgical therapy or a rehabilitation program (12,13). Among the parameters measured during CPET, maximum oxygen uptake (VO2max), the highest attainable rate of transport and use of oxygen by the body during intense exercise, is considered the standard expression of exercise capacity (14).

The aim of this study was to objectively evaluate the cardiorespiratory fitness and physical exercise capacity and response to anti-TNF alpha therapy in patients with AS. The AS patients performed CPET with an incremental ramp protocol to the limit of tolerance before and 4 months after starting anti-TNF therapy.

## 2. Materials and methods

Twenty-eight patients with active AS (BASDAI score of 4 or above) meeting the modified New York diagnostic criteria, with no peripheral joint involvement, aged 18–50 years and who had not previously received anti-TNF alpha therapy, were enrolled in the study. Patients were assessed in terms of the study parameters before anti-TNF alpha therapy and 4 months after commencing therapy. An equivalent number of healthy individuals matched in terms of age, sex, body mass index (BMI), and smoking status were enrolled as the control group. Voluntary consent forms were obtained from the patient and control groups. Patients with exercise intolerance, heart or lung disease, or peripheral joint involvement were excluded. Approval for the study was granted by the Karadeniz Technical University Medical Faculty Local Ethical Committee.

### 2.1. Evaluation parameters 

Patients’ age, sex, BMI, smoking status, duration of disease, delay in diagnosis, and length of drug use were recorded. Evaluation of the patient group before treatment and at the 4th month of treatment and that of the control group was performed by the same clinician (SBK). Chest expansion, the Bath Ankylosing Spondylitis Metrology Index (BASMI), the Bath Ankylosing Spondylitis Disease Activity Index (BASDAI), the Bath Ankylosing Spondylitis Functional Index (BASFI), and the Ankylosing Spondylitis Quality of Life Questionnaire (ASQoL) were used. Routine full blood count, erythrocyte sedimentation rate (ESR), and C-reactive protein (CRP) values were measured concomitantly with clinical evaluation.

### 2.2. Respiratory function test (RFT) 

RFTs were performed using a Koko Legend ergospirometer. The device was recalibrated before each test. Calibration was regarded as valid when a margin of error of less than 3% had been established, as recommend by the American Thoracic Association. The temperature and humidity in the room where the exercise test was performed were adjusted to the weather conditions. Patients were given a detailed explanation of the respiratory maneuvers they would need to perform during the RFT, and dynamic tests were then performed. Forced expiratory volume in 1 s (FEV1), forced vital capacity (FVC), and FEV1/FVC values were thus determined. Measurements were repeated so that the difference between three tests should be less than 10%, and the best value automatically determined by the computer was recorded.

### 2.3. Cardiopulmonary exercise test (CPET) 

Maximum oxygen consumption (VO2max) is widely regarded as the scale that best indicates cardiovascular performance and aerobic exercise capacity. During exercise, oxygen consumption (VO2) rises in parallel with work load until a plateau is reached. This plateau represents the point at which VO2 (mL/kg per min or L/min) will rise no further despite an increasing work load and shows the individual’s VO2max (or aerobic capacity) value. Measurement of VO2 is very useful in objectively identifying the physical work performed by an individual. The main factors affecting VO2max are age, sex, exercise habits, and cardiac status. Women have less muscle mass, lower hemoglobin levels, and lower blood volume and cardiac output than men, and their VO2max values are lower in association with these factors. VO2max will be high in individuals with high physical activity levels.

 All exercise tests were performed in the morning hours (0900–1100 hours), immediately after diagnosis of active disease state and 4 months after initiation of anti-TNF alpha therapy. On the exercise test day, patients were advised to dress comfortably, not to eat or smoke for 3 h beforehand, to drink water so as to ensure normal hydration, to avoid any excessively tiring exercise apart from routine activity, and to sleep for 6 h the night before the test (12). A bicycle ergometer, the most commonly employed modality, was used for the exercise test. The test was performed for every volunteer using bicycle ergometry with a gradually increasing work rate (loading test). The exercise test, in which work rate is gradually increased, began with a warm-up period of 5 min at a work rate of 20 W. At the end of the warm-up period, the work rate was increased by 15 W a minute (5 W/20 s) in a computer-controlled manner. The increase varies according to the health status of the individual and was maintained until each subject could no longer rotate the pedals (not falling below 40 rpm), or in other words until the patient’s ‘exercise potential’ with maximum effort was exhausted. Individuals’ maximal effort capacities (Wmax, watts) and aerobic and anaerobic work capacities were determined with this test. Patients’ cardiac parameters were monitored during exercise using 12-derivation ECG (VIASYS Healthcare, USA). Pulmonary gas exchange parameters were measured using a metabolic gas measurement device, breath by breath, at every respiration (Master Screen CPX Jaeger, Germany). Respiratory parameters were assessed with breath by breath calculation measured using a turbine volume meter. Metabolic (energy-substrate use, RQ), cardiovascular (heart rate, O2 pulse), and separate assessments for aerobic and anaerobic exercise regions were performed during this test. No condition requiring early discontinuation occurred in the exercise test. All volunteers achieved maximal exercise test criteria. No complication was observed during or after the test (15). 

### 2.4. Statistical analysis

SPSS 13.0 for Windows was used for data analysis. The Kolmogorov–Smirnov test was used to assess whether numerical data were normally distributed. Student’s t-test was used for two-group comparison of normally distributed data and the Mann–Whitney U test was used for nonnormally distributed data. The chi-square test was used for nominal data. The paired t-test was used for pre- and postdrug evaluation for parametric data and the Wilcoxon test for nonparametric data. Pearson correlation analysis was used to determine correlation between numerical variables. Significance was set at P < 0.05.

## 3. Results

The patient population consisted of 28 individuals (19 males). Mean age of the patients was 37 ± 9.1 years, and that of the control group was 35 ± 6.1 years (P = 0.331). Mean BMI in the patient group was 28.9 ± 5.3, and it was 27.3 ± 3.7 (P = 0.199) in the control group. There was no statistically significant difference between the groups in terms of sex and smoking status (P > 0.05). Mean duration of disease was 8.9 ± 7.6 years. Sixteen patients (57.1%) used infliximab, 7 (25%) etanercept, and 5 (17.9%) adalimumab. At the initial stage of the study, cardiopulmonary exercise test revealed that VO2peak (mL/kg/min), maximum work load (W), duration of exercise (min), and heart rate reserve (number of beats) were all significantly lower in AS patients than in the controls (Table 1). Assessments at the 4th month after initiation of anti-TNF alpha therapy revealed a significant improvement in all AS-related activity parameters, functional indices, and quality of life. Patients with AS exhibited significantly lower aerobic exercise capacity (VO2peak: 21.2 ± 5.5 vs. 27.2 ± 6.6 mL/kg/min, P = 0.001), maximum power output (110.4 ± 34.8 vs. 153 ± 39.8 W, P = 0.0001), and exercise duration (16.3 ± 2.6 vs. 19.6 ± 2.9 min, P = 0.0001) than the controls. Significant improvements were observed in the objective exercise capacity scales of VO2peak (mL/kg/min) (21.2 ± 5.5 vs. 24 ± 5.5, P = 0.001), maximum work rate (110.4 ± 34.8 *vs. *132.9 ± 37.2 W, P = 0.0001), and duration of exercise (16.3 ± 2.6 vs. 17.6 ± 2.9 min, P = 0.001). The heart rate reserve values were also increased (35.4 ± 16.9 vs. 16.6 ± 9.7, P < 0.0001) (Table 2). A positive correlation was determined between VO2peak values, showing patients’ oxygen consumption, and maximum work load, and a negative correlation was determined between VO2peak and duration of disease, age, BMI, ASQoL, and heart rate reserve. A significant negative correlation was also observed between heart rate reserve and maximum work load (r = –0.389), FVC (r = –0.4), and FEV (r = –0.416).

**Table 1:** Patients’ pretreatment and control group exercise test and SFT parameters.

	AS (n = 28)	Control (n = 28)	P-value
VO2peak (mL/kg/min)	21.2 ± 5.5	27.2 ± 6.6	0.001
Maximum work capacity (W)	110.4 ± 34.8	153 ± 39.8	0.0001
Duration of exercise (min)	16.3 ± 2.6	19.6 ± 2.9	0.0001
Heart reserve rate (number of beats)	35.4 ± 16.9	16.6 ± 9.7	0.0001
FVC (%)	94.2 ± 15.4	104.8 ± 14.6	0.011
FEV1 (%)	98.8 ± 14.9	108.8 ± 11.6	0.007
FEV1/FVC	87.6 ± 4.9	88.6 ± 6.2	0.719

VO2: Oxygen consumption, FVC: forced vital capacity, FEV1: forced expiratory volume in the 1st second.

**Table 2:** Pretreatment and 4th month clinical and laboratory parameters of patients with ankylosing spondylitis.

	Pretreatment (n = 28)	Posttreatment (n = 28)	P-value
Chest expansion (cm)	3.3 ± 1.3	4 ± 1.6	0.0001
Modified Schober test (cm)	13 ± 1.7	13.8 ± 1.6	0.002
BASDAI	6.1 ± 1.4	2.9 ± 1.4	0.0001
BASMI	8.2 ± 2	6.9 ± 1.8	0.0001
BASFI	4.7 ± 2.7	2.4 ± 2.4	0.0001
ASQoL	11.8 ± 4.6	4.9 ± 4.2	0.0001
ESR (mm/s)	39.2 ± 23	15.5 ± 13.7	0.0001
CRP (mg/dL)	1.5 ± 1.1	0.5 ± 0.7	0.0001
VO2peak (mL/kg/min)	21.2 ± 5.5	24 ± 5.5	0.001
Maximum work capacity (W)	110.4 ± 34.8	132.9 ± 37.2	0.0001
Duration of exercise (min)	16.3 ± 2.6	17.6 ± 2.9	0.001
Heart rate reserve (number of beats)	35.4 ± 16.9	16.6 ± 9.7	0.0001
FVC (%)	94.2 ± 15.4	95.8 ± 13.8	0.478
FEV1 (%)	98.8 ± 14.9	100.0 ± 14.8	0.575
FEV1/FVC	87.9 ± 7.0	87.6 ± 5.3	0.967

BASDAI: Bath Ankylosing Spondylitis Disease Activity Index, BASMI: Bath Ankylosing Spondylitis Metrology Index, BASFI: Bath Ankylosing Spondylitis Functional Index, ASQoL: Ankylosing Spondylitis Quality of Life Questionnaire, ESR: erythrocyte sedimentation rate, CRP: C-reactive protein, FVC: forced vital capacity, FV1: forced expiratory volume in the 1st second; P < 0.05 was regarded as statistically significant.

## 4. Discussion

Our study showed a marked and rapid objective improvement in exercise capacity following anti-TNF alpha therapy. The decrease in effort capacity in patients with AS has two main causes. The first is systemic inflammation and associated pain and fatigue. The second is restrictive-type respiratory compromise due to the effect on the pulmonary parenchyma and thoracic cage (2). Pain, stiffness, fatigue, and physical restriction are the main symptoms in patients with AS, and these all affect quality of life and effort capacity (16). Dagfinrud et al. described serious fatigue in one in three patients with AS (6). Various researchers have shown that fatigue is correlated with disease activity, mental status, and functional capacity and has an adverse impact on quality of life (7–10). Cardiopulmonary exercise testing (CPET) is a noninvasive physiological test widely used to test individuals’ exercise response involving pulmonary, cardiovascular, and neuromuscular systems. This noninvasive, dynamic physiological test permits the clinician to make decisions based on responses of organs at physiological functional limits, not just at rest, by providing submaximal and peak exercise responses. Since CPET provides objective data concerning physiological functional capacity and losses occurring in that capacity due to diseases, and about improvements caused by therapeutic interventions (medical, surgery, and physiotherapy), it is being used in an increasingly broad clinical spectrum (12). Examination of oxygen consumption (VO2peak, mL/kg/min, 21.2 ± 5.5 vs. 27.2 ± 6.6, P = 001), maximum work load (W, 110.4 ± 34.8 vs. 153.0 ± 39.8, P = 0.0001), duration of exercise (min, 16.3 ± 2.6 vs. 19.6 ± 2.9, P = 0.0001), and heart rate reserve (number of beats, 35.4 ± 16.9 vs. 16.6 ± 9.7, P = 0.0001) values, objective data showing effort capacity and cardiopulmonary capacity, and comparison of these between the two groups, revealed statistically significant differences. These findings objectively show that the work capacity of patients with AS was lower than that of the healthy control group. The aim of treatment in AS is to reduce pain, stiffness, and fatigue; to establish and maintain proper posture; and to ensure physical and psychosocial functionality (11). The entry into use of anti-TNF alpha drugs represented a turning point in the treatment of AS. Studies involving anti-TNF alpha have reported significant improvements in quality of life and acute phase responses in addition to BASDAI, BASFI, and BASMI scores (17–19). However, findings regarding the effects on radiographic progression and extraarticular involvements are inconsistent (20–23). Activity scores in our patients improved significantly in a short time. A significant improvement in patients’ BASDAI (from 6.1 ± 1.4 to 2.9 ± 1.4), BASFI (from 4.7 ± 2.7 to 2.4 ± 2.4), BASMI (from 8.2 ± 2 to 6.9 ± 1.8), ASQoL (from 11.8 ± 4.6 to 4.9 ± 4.2), ESR (from 39.2 ± 23 to 15.5 ± 13.7), and CRP (from 1.5 ± 1.1 to 0.5 ± 0.7) values (P = 0.0001) was achieved with anti-TNF alpha therapy, as well as clinical and laboratory benefits and an improved quality of life. Disease-related loss of work capacity is widespread in AS. Twenty-four percent of employed subjects with AS were identified as receiving help from colleagues, and that assistance was observed more in patients doing physical work and during periods of more active disease findings (24). In a study of 21 patients with AS, Çakar et al. determined that loss of productivity in working life was correlated with onset of disease at advanced age, prolonged delay in diagnosis, and various physical impairments such as spinal mobility and hip involvement (10). Anti-TNF alpha therapies reduce inflammation in AS and contribute to better functionality and work capacity. Studies involving fatigue and functional indices in AS generally measure effectiveness in a subjective manner, although the reliability of data of patient origin is controversial. The ASDAS, a questionnaire containing laboratory markers (ESR and CRP), was therefore developed for that reason and began being used in daily practice (25). Fatigue, loss of work capacity, and effort intolerance are some of the most common symptoms in patients with AS. Our study was planned to show the effect on work capacity and effort tolerance and the effect of anti-TNF alpha therapy on those indices, in an objective manner, in addition to activity indices. There are no previous studies on this subject. In terms of objective evidence, our study demonstrated a statistically significant increase in patients’ aerobic capacity (VO2peak, mL/kg/min, from 21.2 ± 5.5 to 24 ± 5.5, P = 0.001), maximum tolerable work rate (W, from 110.4 ± 34.8 to 132.9 ± 37.2, P = 0.0001), and duration of exercise (from 16.3 ± 2.6 min to 17.6 ± 2.9 min, P = 0.001) upon assessment at the 4th month of treatment. No previous studies have been performed using this method. To the best of our knowledge, ours is the first study to show, in an objective manner, that anti-TNF alpha therapies increase aerobic capacity. The limitations of the study are relatively low number of patients involved and the short follow-up time. Further studies involving more subjects and longer periods are now needed. 

In conclusion, in addition to having positive effects on inflammatory parameters and quality of life in patients with AS, anti-TNF alpha therapy can achieve rapid and significant improvement in parameters objectively demonstrating the patient’s cardiopulmonary health status, such as VO2peak, maximum work capacity, and duration of exercise. Considering that these patients received treatment for symptoms before anti-TNF alpha, in addition to confirming that the disease leads to a low work capacity, the low work capacity VO2max also shows that standard treatment before anti-TNF alpha was unable to prevent this effect. However, even over a 3-month period, anti-TNF alpha therapy not only controlled disease activity but also (maybe secondary to this) significantly increased cardiopulmonary exercise capacity. These findings may represent an additional and concrete justification for transition to anti-TNF alpha therapy.
